# Using artificial intelligence methods to study the effectiveness of exercise in patients with ADHD

**DOI:** 10.3389/fnins.2024.1380886

**Published:** 2024-04-23

**Authors:** Dan Yu, Jia hui Fang

**Affiliations:** ^1^The College of Education and Liberal Arts, Adamson University, Manila, Philippines; ^2^Medical Faculty, Ludwig Maximilian University of Munich, Munich, Germany

**Keywords:** ADHD, artificial intelligence, motor information, Random Forest, TCN, ACT-R

## Abstract

Attention Deficit Hyperactivity Disorder (ADHD) is a prevalent neurodevelopmental disorder that significantly affects children and adults worldwide, characterized by persistent inattention, hyperactivity, and impulsivity. Current research in this field faces challenges, particularly in accurate diagnosis and effective treatment strategies. The analysis of motor information, enriched by artificial intelligence methodologies, plays a vital role in deepening our understanding and improving the management of ADHD. The integration of AI techniques, such as machine learning and data analysis, into the study of ADHD-related motor behaviors, allows for a more nuanced understanding of the disorder. This approach facilitates the identification of patterns and anomalies in motor activity that are often characteristic of ADHD, thereby contributing to more precise diagnostics and tailored treatment strategies. Our approach focuses on utilizing AI techniques to deeply analyze patients' motor information and cognitive processes, aiming to improve ADHD diagnosis and treatment strategies. On the ADHD dataset, the model significantly improved accuracy to 98.21% and recall to 93.86%, especially excelling in EEG data processing with accuracy and recall rates of 96.62 and 95.21%, respectively, demonstrating precise capturing of ADHD characteristic behaviors and physiological responses. These results not only reveal the great potential of our model in improving ADHD diagnostic accuracy and developing personalized treatment plans, but also open up new research perspectives for understanding the complex neurological logic of ADHD. In addition, our study not only suggests innovative perspectives and approaches for ADHD treatment, but also provides a solid foundation for future research exploring similar complex neurological disorders, providing valuable data and insights. This is scientifically important for improving treatment outcomes and patients' quality of life, and points the way for future-oriented medical research and clinical practice.

## 1 Introduction

ADHD is a common neurodevelopmental disorder that widely affects children and adults worldwide. Its main characteristics include persistent inattention, hyperactivity and impulsive behaviors, which often have a significant impact on an individual's ability to learn, socialize, and work (Tang et al., 2020). The diagnosis of ADHD is complex and varied and often requires a combination of medical, psychological and behavioral evaluations (Loh et al., 2023). Currently, the exact cause of ADHD is not fully understood, and it is widely believed that a combination of genetics, environmental factors, and variations in brain development play a role (Tan et al., 2023). This complexity makes accurate diagnosis and effective treatment of ADHD a challenge (Amado-Caballero et al., 2020). Traditional diagnosis of ADHD relies on behavioral observations and psychological assessments, but these methods carry the potential for subjective judgments that can lead to diagnostic inconsistencies and accuracy issues (Shoeibi et al., 2023). In addition, due to the diversity of ADHD symptoms and their similarity to other disorders, it is often difficult for a single diagnostic approach to fully capture the full picture of the disease (Berrezueta-Guzman et al., 2021a). Therefore, researchers have been seeking more objective and accurate diagnostic tools.

The analysis of motor information plays a pivotal role in ADHD research and treatment, as hyperactive behavior significantly influences a patient's daily functioning and learning capabilities (Enriquez-Geppert et al., 2019). Motor control issues and hyperactivity, essential for diagnosis and treatment planning, offer insights into behavioral and neurophysiological changes in individuals with ADHD (Chen et al., 2020; Slobodin et al., 2020; Berrezueta-Guzman et al., 2021b). Movement tracking technologies and comprehensive analysis of motor behaviors can elucidate ADHD's neurobiological foundations (Amado-Caballero et al., 2023), enhancing diagnostic accuracy and aiding the development of more effective treatments (Berrezueta-Guzman et al., 2022). Additionally, advancements in Artificial Intelligence (AI) have transformed ADHD diagnosis and treatment strategies, with machine learning techniques uncovering complex patterns in data, facilitating preliminary feature selection and analysis (Moghaddari et al., 2020; Zhang et al., 2021a; Tang et al., 2022). This evolving AI landscape necessitates sophisticated, integrative models for a more nuanced understanding of ADHD (Leontyev et al., 2019; Yeh et al., 2020).

However, challenges remain in harnessing AI for ADHD research, notably in data acquisition, processing, and model comprehensiveness and interpretability. High-quality data collection and processing are critical for reliable research outcomes, but standardized, comprehensive datasets are difficult to obtain due to data diversity, complexity, and privacy concerns (Öztekin et al., 2021). Furthermore, the significant individual variability in ADHD symptoms and behaviors requires models that can integrate various data sources and analytical methods to accurately reflect these differences (Chen et al., 2021), highlighting the need for continued innovation in AI methodologies to address these challenges effectively.

In response to the identified gaps in existing research, we have developed an innovative network model that seamlessly integrates Random Forest, Temporal Convolutional Network (TCN), and Adaptive Control of Thought-Rational (ACT-R) to examine the effects of physical exercise on ADHD patients. This integrated framework is designed to transcend the limitations of traditional methodologies by leveraging the distinct strengths of each component (Speiser et al., 2019), thereby enhancing diagnostic accuracy and efficiency in handling complex ADHD-related data. The Random Forest algorithm, recognized for its prowess in managing high-dimensional data, plays a pivotal role in our model by identifying and isolating key features associated with ADHD symptoms. This process not only aids in refining input data for deeper analysis but also capitalizes on its capability to navigate non-linear and intricate data relationships (Dimov et al., 2020). Concurrently, the TCN model, with its specialization in processing time-series data, adeptly captures the dynamic changes in behavior and physiology characteristic of ADHD, thus offering a nuanced reflection of the patients' behavioral patterns and physiological states over time.

The model performs feature extraction and selection of multi-source data through the Random Forest algorithm to effectively identify key features associated with ADHD symptoms. Next, TCN is used to analyze time-series data from these features to capture behavioral and physiological signals over time. The ACT-R model is used to simulate the cognitive processes of ADHD patients to help predict their behavioral responses and symptom performance. Finally, the results of these analyses are synthesized and optimized for diagnosis and treatment prediction of ADHD using deep learning algorithms. By integrating these three models, our network model is able to provide an in-depth understanding of the behavioral and cognitive characteristics of ADHD patients from multiple dimensions and optimize the diagnosis and treatment prediction of ADHD using deep learning algorithms. This multidimensional and multimodal integrated approach is not only more accurate and effective in dealing with complex ADHD data, but also improves the accuracy of diagnosis and personalization of treatment. In addition, this approach helps to reveal the complex pathological mechanisms of ADHD, providing new perspectives and methods for future research and treatment strategies. This fusion model not only deepens the understanding of ADHD, but also provides a new, more precise and comprehensive analytical tool for clinical practice, which has important application value. In the subsequent sections of this thesis, we will detail our model architecture and experimental results to validate its effectiveness in studying the effects of exercise in patients with ADHD.

The contribution points of this paper are as follows:

- We have successfully developed a novel fusion model that integrates Random Forest, TCN, and ACT-R algorithms. This innovative integration approach has demonstrated outstanding performance in processing ADHD data, particularly in enhancing diagnostic accuracy and understanding the pathophysiology.
- Our research is the first to combine deep learning techniques with cognitive psychology models in the analysis of ADHD, providing a new perspective for the diagnosis and treatment of ADHD. This interdisciplinary approach allows us to gain a deeper understanding of the behavioral and cognitive characteristics of ADHD patients, laying the groundwork for developing more effective personalized treatment strategies.
- Our model has been validated on actual clinical data and has shown efficient computational performance and good scalability. This achievement not only proves the practicality of our model but also provides a reliable reference for applying deep learning and cognitive models in future research on similar complex neurological disorders.

## 2 Related work

### 2.1 DNN for analyzing ADHD patients' response to exercise

Recent endeavors in the realm of ADHD research have seen the application of Deep Neural Networks (DNN) to parse through complex, multidimensional datasets (Baxi et al., 2022), ranging from biometric readings to comprehensive behavioral assessments (Gupta et al., 2022). By harnessing the power of DNN, researchers aim to uncover the nuanced effects that physical activities exert on the ADHD phenotype, hoping to identify patterns that correlate with symptom alleviation or exacerbation (Wang et al., 2024). The capability of DNN to process vast arrays of input data and to learn from these inputs in an unsupervised or semi-supervised manner has opened up new avenues for predicting the outcomes of various therapeutic interventions, including exercise and movement-based therapies.

However, deploying DNN in ADHD research is fraught with challenges. The primary issue revolves around the interpretability of the models. The intrinsic complexity of DNN architectures, while a boon for navigating large data sets, renders the extraction of clear, actionable insights difficult (Ahmadi et al., 2021). Clinicians and therapists seeking to apply these findings are often met with a gap between statistical significance and practical applicability. Moreover, the reliance on extensive computational resources for data processing and model training limits the accessibility of DNN methodologies, particularly in resource-constrained research environments. The demand for vast, meticulously annotated data sets further complicates research efforts (Hernández-Capistran et al., 2023), given the inherent variability in ADHD manifestations across individuals and the ethical considerations tied to patient data privacy.

### 2.2 SVM in identifying ADHD biomarkers from physical activity data

The use of Support Vector Machines (SVM) in analyzing behavioral data presents a focused approach to understanding ADHD, especially in the context of physical activity interventions (Mohd et al., 2022). SVM's robust classification capabilities allow for the distinction between ADHD-affected individuals and their neurotypical peers based solely on quantified behavioral metrics derived from physical activity patterns (Wang et al., 2018). Such analyses are instrumental in pinpointing potential behavioral biomarkers for ADHD, facilitating a deeper comprehension of the disorder's external manifestations and the ways in which targeted physical interventions might ameliorate or modify these behaviors.

Despite the strengths of SVM in classification tasks, the model's application in ADHD research is not devoid of limitations. The necessity for labeled data poses a significant bottleneck (Chen et al., 2023), especially in early-stage research where diagnostic ambiguity prevails. Additionally, SVM models, traditionally linear, may struggle with the complex, non-linear behavioral patterns characteristic of ADHD, even though kernel methods can offer some mitigation (Eslami et al., 2021). The focus on behavioral data, to the exclusion of neurophysiological or cognitive data, might also narrow the scope of findings, potentially overlooking multifaceted aspects of ADHD symptomatology.

### 2.3 CNN for processing EEG data in ADHD exercise studies

Convolutional Neural Networks (CNN) have revolutionized the analysis of neurophysiological data, such as EEG, offering fresh perspectives on the neurological aspects of ADHD (TaghiBeyglou et al., 2022). The application of CNN to EEG data pre- and post-physical activity interventions has shed light on the neurophysiological shifts that might underlie observed behavioral changes in ADHD patients. CNN's adeptness at detecting spatial hierarchies in data makes it uniquely suited to identifying patterns within the complex signals characteristic of EEG recordings (Ribas et al., 2023), providing a conduit for exploring the neurobiological impact of exercise on individuals with ADHD.

The implementation of CNN in the study of ADHD through neurophysiological data is not without challenges. The model's sensitivity to the specificities of the training data raises concerns about overfitting (Delvigne et al., 2021), particularly acute in neurophysiological studies where sample sizes are often limited. The preprocessing required to adapt EEG data for CNN analysis is both intricate and labor-intensive, risking the introduction of bias or the loss of critical information (Sawangjai et al., 2019). Moreover, the complexity of CNN outputs complicates their translation into clinically relevant insights, presenting an ongoing challenge for bridging the divide between advanced AI-driven analyses and actionable treatment strategies for ADHD.

By elaborating on these studies, we gain a nuanced understanding of the current landscape of AI in ADHD research concerning physical activity, acknowledging the progress made and the hurdles that lie ahead. This comprehensive view serves as a critical stepping stone for future investigations aimed at harnessing AI's full potential in this domain.

## 3 Materials and methods

### 3.1 Overview of our network

In this study, we have developed an integrated model combining Random Forest, Temporal Convolutional Network (TCN), and Adaptive Control of Thought-Rational (ACT-R) to investigate the effects of physical activity in patients with ADHD.

We developed a comprehensive model that integrates the strengths of Random Forest, TCN and ACT-R to cope with the complexity of ADHD. Random Forest is crucial for feature selection and extraction. It processes the initial input data, identifying and isolating key features that are most relevant to ADHD symptoms and motor activities. The strength of Random Forest lies in its ability to handle high-dimensional data and uncover complex, non-linear relationships, making it ideal for the initial analysis stage. TCN serves as the core component for analyzing time-series data, particularly motor monitoring and neurophysiological data. Its architecture, designed to handle sequential data, captures temporal dependencies and dynamic changes in ADHD patients' behavior and physiological responses. TCN's effectiveness in our model stems from its deep, dilated convolutional structure, enabling detailed analysis of intricate time-related patterns. ACT-R is utilized to simulate and interpret the cognitive processes of ADHD patients. This model integrates the outputs from the Random Forest and TCN, providing a cognitive perspective to the analysis. It helps in understanding how ADHD affects cognitive functions and how physical activities might influence these cognitive patterns.

**Figure 1:**
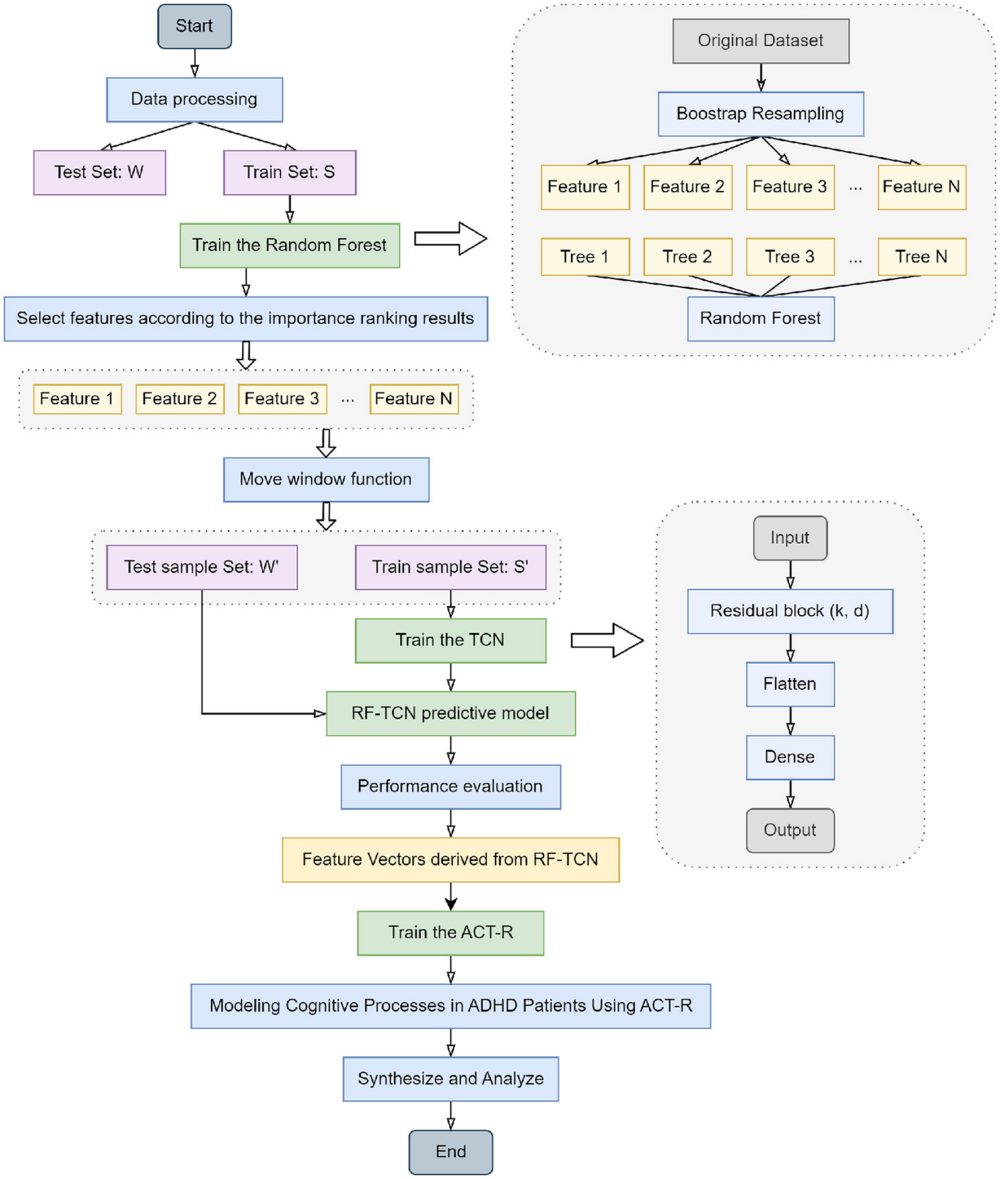
Flowchart of the overall structure of our model.

The workflow of our integrated model, detailing the collaborative functions of Random Forest, TCN, and ACT-R in the context of ADHD physical activity research, is systematically illustrated in the flowchart presented in Figure 1. In constructing our integrated network model, we began with the data processing of the original dataset, which included Bootstrap resampling to ensure consistency of data across the training and test sets. The Random Forest algorithm was trained on dataset S, selecting key features based on importance rankings. These features were then transformed into time series datasets S' and W' using a moving window function, preparing them for TCN training. This process readied TCNs for training by sliding a fixed-size window along the time axis of the dataset and capturing local data features within each window. The generated time series datasets were then fed into the TCN, which was trained using its residual blocks defined by kernel size *k* and dilation coefficient d to build the RF-TCN predictive model. Data processed through these residual blocks passed through Flatten and Dense layers to generate the final output.

Upon the training and performance evaluation of the RF-TCN model, we proceeded to integrate the ACT-R model. The input to the ACT-R model consists of feature vectors derived from the Random Forest and Temporal Convolutional Network (RF-TCN) model. These vectors encapsulate the significant features related to ADHD symptoms' behaviors and physiological responses, pinpointed through our initial analyses. Utilizing this input, the ACT-R model was further trained to simulate the cognitive processes of ADHD patients. This process aimed at blending the time series analytical capabilities of the RF-TCN model with the cognitive simulations facilitated by the ACT-R framework. The output of the ACT-R model encompasses predictions on cognitive states and potential behavioral responses of ADHD patients to various exercise regimens. By providing a comprehensive analysis of the behavioral and cognitive patterns of ADHD patients under physical intervention, this output is invaluable for understanding how specific exercises can influence cognitive functions and behavioral patterns in patients with ADHD. This integrated approach not only enhances our ability to understand and evaluate the impact of physical activity on ADHD patients but also lays the groundwork for further personalized treatment approaches, aiming to tailor individualized exercise-based treatment plans based on the predictive insights generated by the ACT-R model.

The significance of our model lies in its multifaceted approach to understanding ADHD. By combining the strengths of Random Forest, TCN, and ACT-R, our model offers a comprehensive analysis of ADHD patients' motor activities and their cognitive implications. This integrated approach allows for a deeper understanding of how physical activity affects ADHD patients, not just in terms of immediate motor responses but also in long-term cognitive and behavioral changes. The model's ability to process complex data and provide insights into the temporal dynamics of ADHD presents a significant advancement in researching effective treatment and management strategies for ADHD, particularly in the realm of physical interventions.

To ensure the trustworthiness and transparency of our AI model, we incorporated an interpretability and reliability analysis into our methodology. For interpretability, we utilized SHapley Additive exPlanations (SHAP) values to quantify the impact of each feature on the model's predictions. This approach helps in identifying the most influential factors contributing to the model's decision-making process. Additionally, to assess the reliability of our model, we employed a rigorous cross-validation technique, along with an external validation on a separate dataset, ensuring the model's robustness and its capability to generalize across different populations.

The interpretability analysis revealed that certain features, such as the duration and intensity of exercise, played a significant role in the model's predictions regarding the effectiveness of exercise in ADHD patients. SHAP value plots highlighted these features' positive influence on the model's confidence in predicting improvement in ADHD symptoms, offering insights into how exercise routines can be optimized for therapeutic purposes.

The reliability analysis, conducted through 10-fold cross-validation and further validated on an external dataset, demonstrated consistent accuracy levels, underscoring the model's robustness. The slight variations observed across different folds were within acceptable limits, indicating the model's capability to generalize and perform reliably in diverse settings.

### 3.2 Random Forest

Random Forest is a machine learning classifier composed of multiple decision trees. It is capable of handling classification, regression, and dimensionality reduction problems (Borup et al., 2023). In a Random Forest, each decision tree operates independently and without correlation to others (Sheykhmousa et al., 2020). For classification tasks, each tree classifies the test sample, and the final category is determined by the mode of the outputs from the forest, essentially using a voting mechanism to decide the category of the test sample. For regression tasks, the final result is the average of the outputs from all trees. Compared to a single decision tree, Random Forest exhibits a stronger tolerance to outliers and noise and shows better performance in both prediction and classification (Cheng et al., 2019).

A decision tree is a commonly used algorithm for classification and regression (Maji and Arora, 2019). It constructs a tree-like structure by dividing the dataset into different subsets, where each node represents a feature, each branch represents a value of that feature, and each leaf node represents a category or a value. In building a decision tree, the optimal feature for splitting must be selected, which necessitates the concept of entropy. Entropy is a measure of the uncertainty of a dataset (Li et al., 2021); the greater the entropy, the higher the uncertainty. In decision trees, we aim to select the optimal feature that minimizes the entropy of the subsets post-split, thereby enhancing the accuracy of the decision tree. Therefore, entropy can be used to measure the information gain of each feature, aiding in the selection of the optimal feature.

The entropy in a decision tree can be calculated using Equation 1:


(1) $$\begin{array}{l} H\left(D\right)=-\overset{n}{\underset{i=1}{\sum }}p_{i}log_{2}p_{i} \end{array}$$

Here, *H*(*D*) denotes the entropy of dataset *D*, *n* is the number of categories in *D*, and π represents the proportion of samples of the *i*th category in *D*. To calculate entropy, we compute the proportion of each category in the dataset and substitute these into the formula.

In decision trees, it is also necessary to calculate the information gain of each feature for optimal splitting. Information gain can be calculated using Equation 2:


(2) $$\begin{array}{l} Gain\left(D,a\right)=H\left(D\right)-\overset{V}{\underset{v=1}{\sum }}\frac{\left|D^{v}\right|}{\left|D\right|}H\left(D^{v}\right) \end{array}$$

where *Gain* (*D, a*) denotes the information gain of dataset D on feature a, *H*(*D*) is the entropy of dataset *D*, *V* represents the number of values for feature *a*, *D*^*v*^ is the subset of samples where feature a has the value *v*, |*D*^*v*^| is the number of samples in *D*^*v*^, and |*D*| is the number of samples in dataset *D*. When calculating information gain, we compute it for each feature in the dataset and select the feature with the highest information gain for splitting.

The process of Random Forest involves several key steps. Initially, it includes a random sampling process where the model samples both rows and columns from the input data. For row sampling, it employs a bootstrap method, meaning that the sampled dataset may contain duplicate samples. If the input sample size is *N*, then the sampled dataset will also have *N* samples. This approach ensures that each tree in the training phase does not use all the samples, reducing the likelihood of over-fitting. For column sampling, out of M features, a subset of m features (where m < < M) is selected. Following this, decision trees are constructed using a complete splitting method, where each leaf node either cannot be further split or contains samples belonging to the same category. Unlike many decision tree algorithms that involve a crucial step of pruning, Random Forest does not require this due to the randomness introduced in the two sampling processes, thereby preventing over-fitting even without pruning.

**Figure 2:**
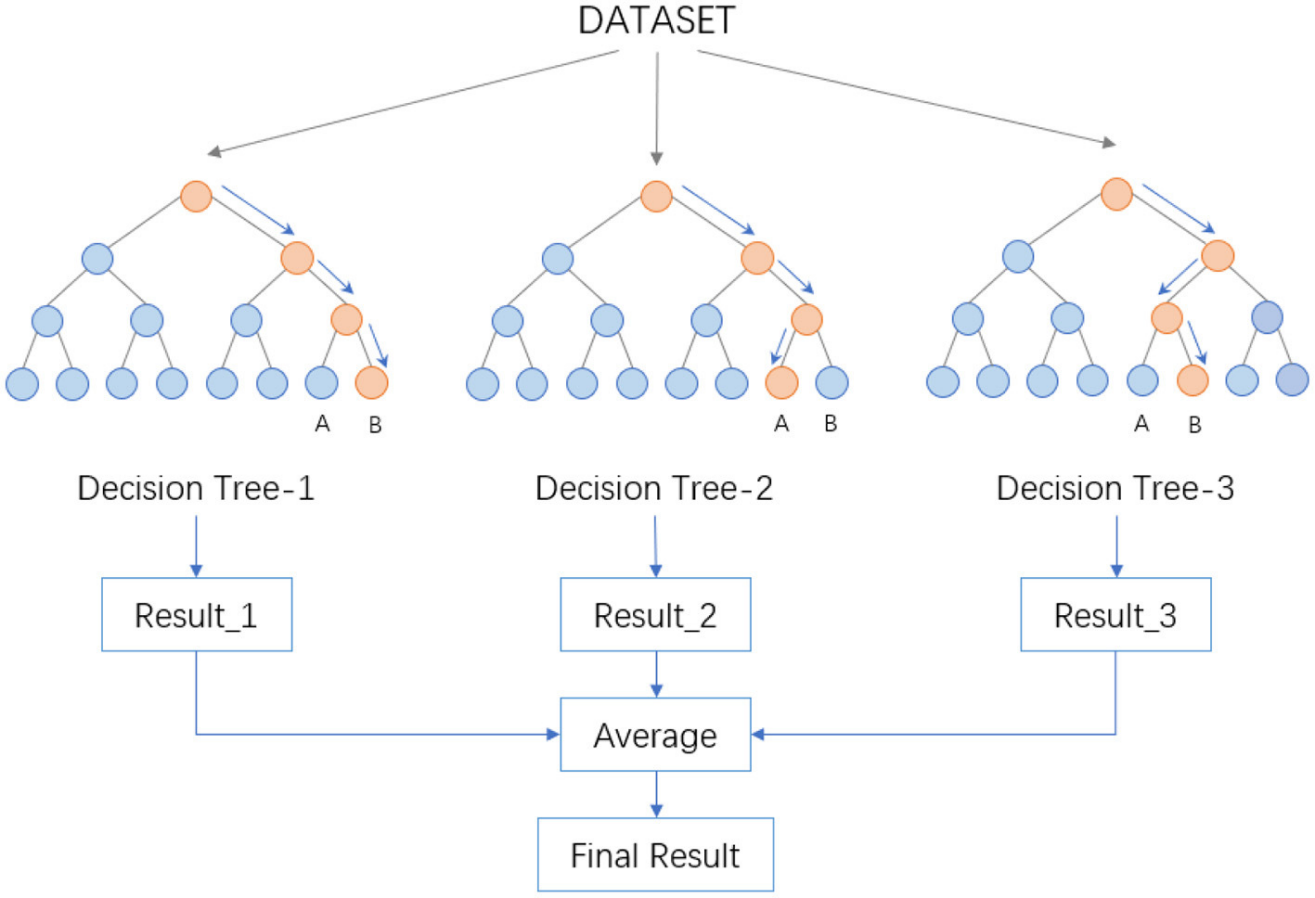
Data processing flow in random forest.

The procedure then involves drawing a specific number of samples from the training set randomly to form the root node samples for each tree. During the construction of the decision trees, a set number of candidate attributes are randomly selected, and the most suitable one is chosen as the splitting node. Once the Random Forest is built, for a test sample, each decision tree produces either a class output or a regression output. In classification problems, the final category is determined through a voting mechanism among the decision trees, while for regression problems, the final result is the average output of all the trees. As depicted in the Figure 2, suppose a Random Forest consists of three decision trees, with two trees classifying a sample as Category B and one as Category A, the Random Forest would classify the sample as Category B.

The randomness in Random Forest is reflected in two aspects. Firstly, it's exhibited in the randomness of sample selection, where a certain number of samples are randomly drawn from the training set with replacement to construct sub-datasets. These sub-datasets are of the same size as the original dataset, and elements within them can be repeated. Secondly, the randomness is in the selection of attributes. During the construction of each decision tree, a certain number of candidate attributes are randomly selected, from which the most suitable attribute is chosen for the splitting node. This process ensures diversity among the trees in the Random Forest, thereby enhancing the classification performance.

In our research, Random Forest, as a core tool, works in conjunction with the ACT-R model and the TCN model, playing a vital role. It employs an ensemble learning approach to comprehensively process and analyze data and insights obtained from both the ACT-R and TCN models. The ACT-R model is used to simulate the cognitive processes of ADHD patients, particularly during physical activities, while the TCN model primarily handles time-series data related to movement, such as motion monitoring or neurophysiological data.

The primary task of the Random Forest is to integrate and analyze these diverse data sources. Through its multitude of decision trees, Random Forest is capable of effectively handling high-dimensional and complex datasets, which is crucial for our research. It aids in identifying key factors affecting ADHD patients from multiple dimensions and enables precise predictions. Through the analysis conducted by Random Forest, we gain a deeper understanding of the potential cognitive and behavioral impacts of physical interventions on ADHD patients and predict the potential effects of different types of physical interventions on various patient groups. These insights are invaluable for designing more effective treatment plans and intervention measures, providing us with data-driven decision support.

Especially in the context of studying the impact of physical activities on ADHD patients, the application of Random Forest is particularly significant. It integrates various data sources, such as neuroimaging data and behavioral observation data, offering a comprehensive analytical perspective for our research. By analyzing different feature combinations, Random Forest helps to reveal the effects of physical interventions on the cognitive and behavioral patterns of ADHD patients. Its high-accuracy predictive and classification capabilities can also be used to assess the effectiveness of physical interventions for different types of ADHD patients and identify which patients may benefit most from specific types of physical activities. Thus, Random Forest becomes a powerful tool in addressing this complex issue.

### 3.3 Temporal convolutional networks

The Temporal Convolutional Network (TCN) is a neural network architecture specifically designed for processing time series data, with its core feature being the utilization of one-dimensional convolutional layers for handling such sequential data. A key characteristic of the TCN is causal convolution, ensuring that the model uses only the current and previous data points for predictions, effectively preventing the leakage of future information. This attribute is crucial for ensuring the accuracy and reliability of the model's predictions. Additionally, TCN incorporates a design with residual connections, similar to those used in ResNet. These residual connections help address the issue of vanishing gradients common in training deep networks (Gao et al., 2023), thereby enhancing the efficiency and stability of model training. This is particularly significant when dealing with complex time series data. Through its unique structure and functions, the TCN provides an effective method for understanding and analyzing time series data, making it particularly suitable for applications involving long-term data dependencies and complex dynamic patterns.

**Figure 3:**
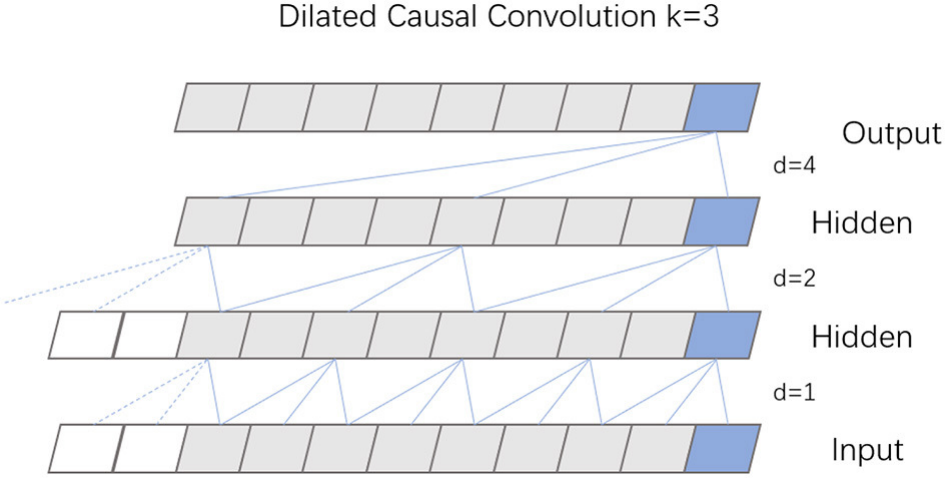
Structure diagram of dilation causal convolution.

TCN, built upon the principles of CNN, features a dilated causal convolution architecture that maintains equal lengths for both input and output. The specific structure of this dilated causal convolution is depicted in Figure 3. This design choice in TCN, emphasizing dilated convolutions, enables the model to efficiently handle sequential data while preserving the temporal sequence length from input to output.

Causal convolution is designed to exclusively consider present and past data points, disregarding any future information. This approach means that for any given time *t* in the output sequence, the result is influenced solely by the input sequence's elements at time *t* and earlier, thus preserving the integrity of historical data. Expanding on this concept, dilated convolution incorporates a dilation coefficient *d*, which dictates the interval at which the input is sampled, thereby enlarging the receptive field of each convolutional layer. The extent of this dilation, and consequently the sampling rate, hinges on the value of *d*. Typically, as a network deepens, the dilation coefficient *d* increases exponentially, often doubling with each added layer. To maintain uniformity in the size of the data through the network layers, and to ensure the output layer matches the width of the input layer, zero padding is employed within each layer of the dilated causal convolution.

**Figure 4:**
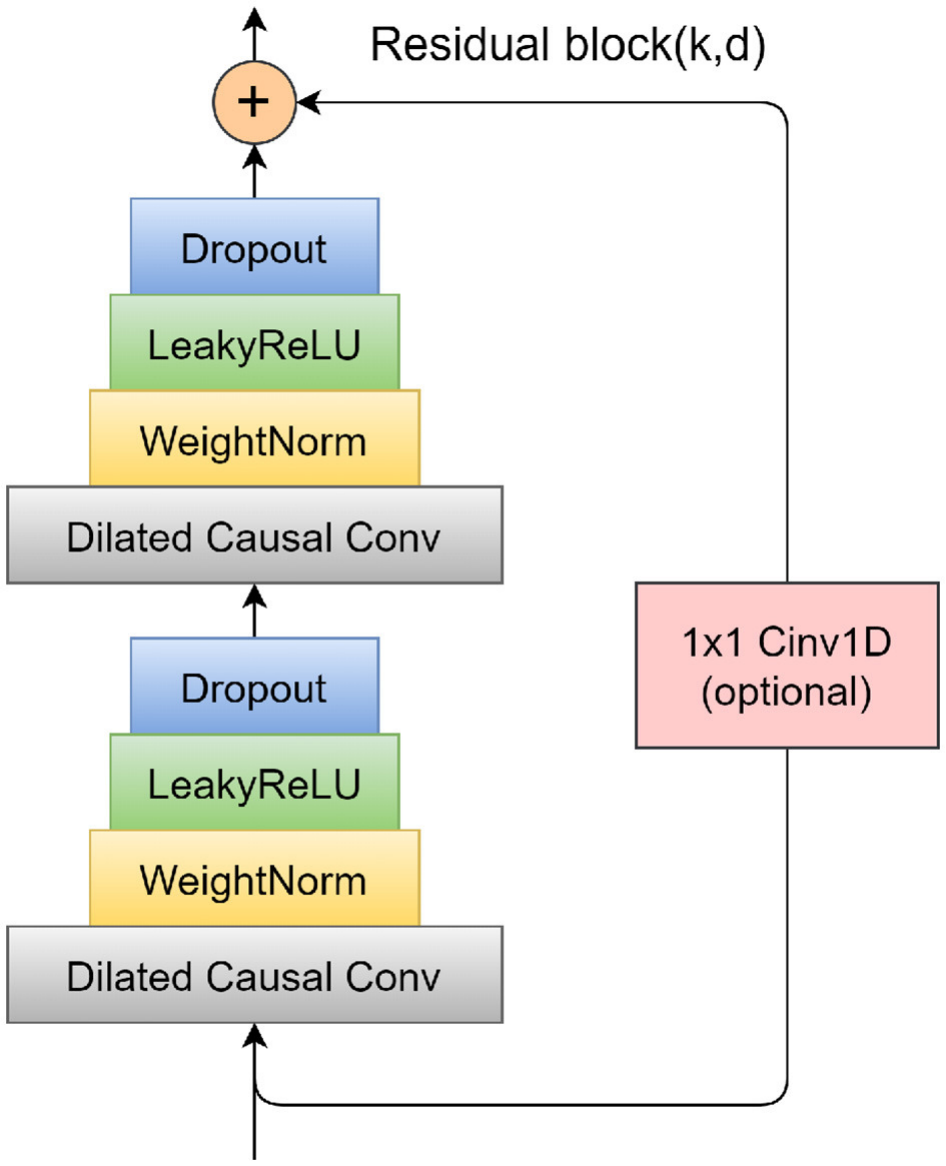
Schematic diagram of residual block.

To mitigate the problems of vanishing and exploding gradients that often arise in overly deep network structures, TCN incorporates a specifically designed residual block. This block consists of two layers of dilated causal convolution, complemented by a non-linear mapping arrangement that incorporates both a WeightNorm and a Dropout layer. A detailed illustration of this residual block structure is presented in Figure 4. The WeightNorm layer functions to standardize the weights within the network layer, thereby streamlining the training process, while the Dropout layer plays a crucial role in preventing overfitting. This configuration equips the TCN with the combined attributes of CNNs and RNNs. Its uncomplicated yet adaptable structure allows for parallel processing of input sequences, which significantly cuts down on both the memory usage and time required for network training. In medical and health research applications, TCN's capability to forecast long-step outputs from complex feature sets demonstrates a notable advantage over traditional models like LSTM and GRU.

In our experiments, the TCN works in conjunction with the Random Forest and ACT-R models, providing support for research into the impact of physical activity on patients with ADHD (Tian et al., 2023). The primary role of the TCN in this model combination is to process and analyze time series data, such as movement monitoring and neurophysiological data. These data are key to understanding the dynamic changes in the behavior and physiological responses of ADHD patients, and the TCN, with its deep and dilated convolutional structure, effectively captures these complex temporal dependencies. The results of the TCN analysis provide a rich feature input for the Random Forest and, when combined with the output from the ACT-R model, offer us a comprehensive perspective for understanding the cognitive and behavioral patterns of ADHD patients under physical activity interventions.

The application of TCN has demonstrated its significance in analyzing the impact of physical activity on patients with ADHD. It not only provides a deep understanding of the immediate effects of physical interventions on the behavior of ADHD patients but also plays a crucial role in capturing the long-term effects of such interventions. Through in-depth analysis by TCN, we can uncover how physical interventions affect the daily behavior and cognitive patterns of ADHD patients, which is essential for accurately assessing the effectiveness of physical activity as a therapeutic approach. The analysis by TCN reveals both the immediate and long-term effects of physical activity on patients' cognition and behavior, and provides crucial data support for designing more personalized and effective treatment plans. This profound analysis and understanding of time series data offer a new perspective and approach for exploring the role of physical activity in treating ADHD, providing significant scientific evidence for enhancing treatment effectiveness and improving patients' quality of life. It also offers valuable data and insights for future research.

### 3.4 ACT-R

The ACT-R (Adaptive Control of Thought—Rational) model is a cognitive architecture specifically designed to simulate human cognitive processes (Fisher et al., 2020). This model is predicated on the assumption that human cognition is comprised of multiple interacting subsystems, each responsible for processing different types of information, such as visual and motor information. The core of the ACT-R model lies in decomposing the cognitive process into a series of modular components, each dedicated to processing specific types of information. This includes modules for storing long-term memory (Zhang et al., 2021b), buffers for processing short-term memory, and a decision center that guides behavior based on information from various modules. Additionally, ACT-R incorporates several distinct modules, each simulating specific human cognitive functions, such as thinking and decision-making processes, thereby facilitating the study and understanding of cognitive psychology phenomena.

**Figure 5:**
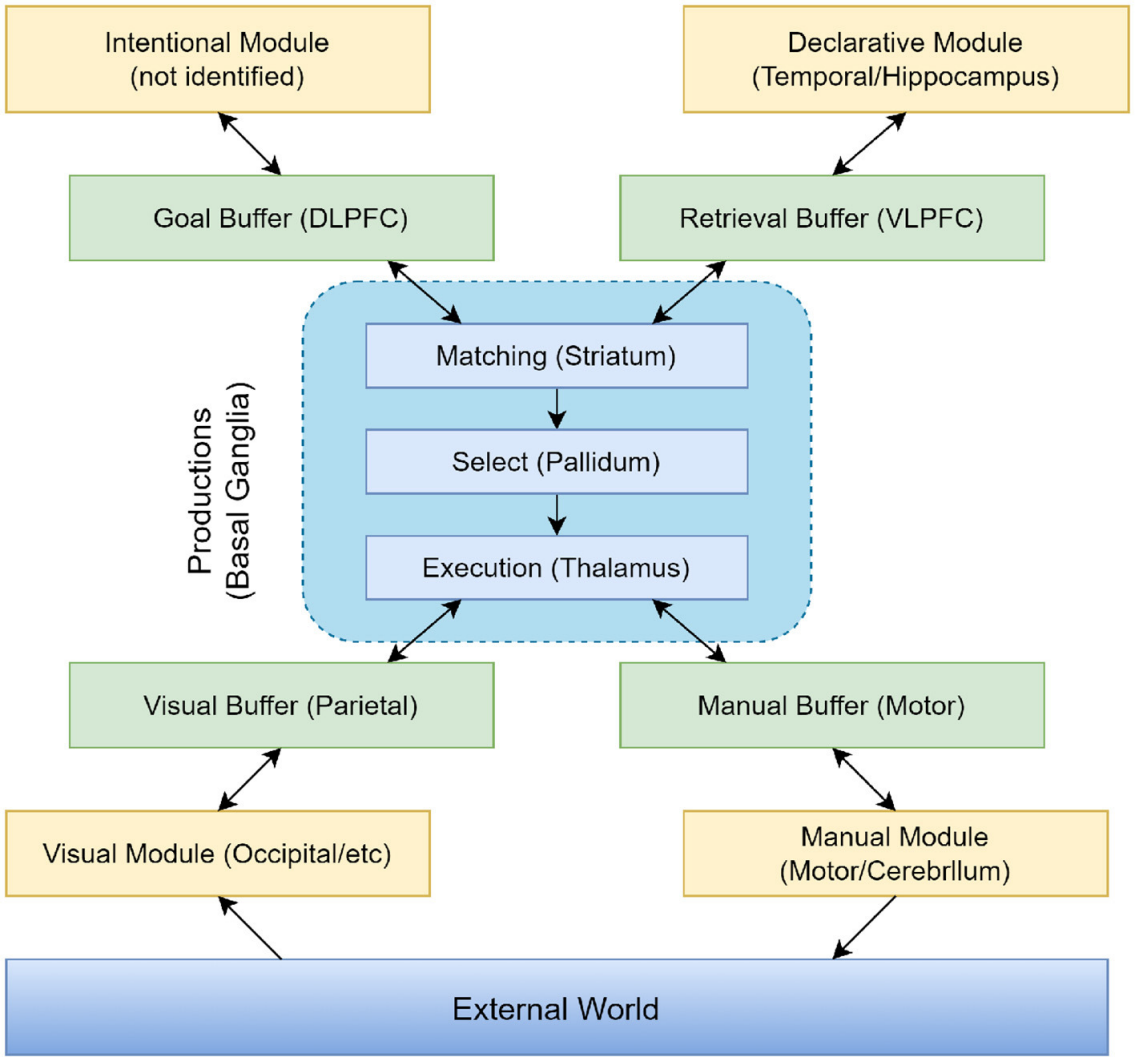
Information organization in ACT-R 5.0.

The ACT-R system is a hybrid cognitive architecture consisting of both symbolic and sub-symbolic systems. As can be seen in Figure 5, the symbolic system is composed of several modules, with a procedural module at its core. This procedural module connects the various modules into a cohesive whole, functioning similarly to a model driven by a production system, where procedural rules in the module manipulate the buffers of different modules. The sub-symbolic system, although not explicitly represented in visualizations, controls the internal operations of modules in the symbolic system through mathematical methods. This structure allows the ACT-R model to simulate human cognitive processes with greater precision and comprehensiveness.

In the ACT-R model, different modules assume various functions and tasks. The Intentional Module (also known as the Goal Module) serves as the executive control center, responsible for planning and controlling behavior. It determines the goals of the current task and coordinates the activities of other modules to achieve these goals. The Declarative Module acts as a repository for storing facts, rules, and conceptual knowledge, supporting the model in accessing and retrieving information from long-term memory during decision-making and problem-solving processes. The Visual Module processes sensory input, simulating the human visual processing system. This module is responsible for perceiving and understanding visual information, such as objects, scenes, and symbols. The Manual Module enables the model to perform manual actions, such as moving and grasping objects. It controls the model's movements and interactions, simulating the execution of physical actions. The Production Module (also referred to as the Procedural System) is one of the core components of ACT-R, representing knowledge and decision-making. It includes production rules that describe condition-action pairs. When specific conditions are met, these production rules are triggered, executing corresponding actions and simulating the decision-making process in cognitive tasks.

In our proposed integrated model, the ACT-R model plays a crucial role in providing a deep understanding of the cognitive processes in ADHD patients. This encompasses how they process information, make decisions, and respond behaviorally to various stimuli, such as physical interventions. By leveraging the feature vectors derived from the RF-TCN model, the ACT-R model utilizes its comprehensive cognitive architecture to simulate these cognitive processes accurately. This architecture is adept at mirroring various cognitive functions, including memory processes, attention, and decision-making, thereby laying the groundwork for understanding the complex behavioral patterns that ADHD patients may exhibit in response to physical intervention.

The integration of the ACT-R model with the TCN's capability in processing time-series data, like motion monitoring data, alongside the advantages of Random Forest in analyzing and integrating multi-dimensional data, culminates in a multi-faceted and multi-layered analytical framework. Through this simulation, the ACT-R model not only learns to associate specific patterns of physical activity with cognitive outcomes in ADHD patients but is also designed to simulate and understand the cognitive decision-making processes. It achieves this by mapping the input features—processed information from the RF-TCN model—to cognitive states that represent ADHD characteristics. This dynamic process enables the ACT-R model to learn the underlying cognitive patterns associated with ADHD, offering valuable insights into how various factors might influence cognitive processes in patients.

Combined, these elements highlight the integrative approach of our research, demonstrating how the ACT-R model's simulation capabilities, when enriched with data from the RF-TCN model, form a comprehensive analytical tool. This tool not only deciphers the intricate cognitive underpinnings of ADHD but also facilitates a nuanced understanding of how physical interventions can be optimized for therapeutic efficacy, based on individual cognitive responses.

The ACT-R model is vital in our experiments because it enables us to comprehend the impact of physical activities on ADHD patients from a cognitive perspective. By simulating the cognitive processes of ADHD patients, we gain a deeper understanding of their responses to physical interventions, including changes at cognitive, emotional, and behavioral levels. This in-depth understanding is crucial for assessing the effectiveness of physical interventions, especially when designing targeted treatment plans and intervention measures. Overall, the ACT-R model provides a unique perspective in our research, complementing the capabilities of TCN and Random Forest in data processing and analysis, and offers a key cognitive dimension to understand the overall impact of physical activities on ADHD patients.

ACT-R is not only a tool for simulating human cognitive processes but also a bridge linking the inner cognitive processes and external behavioral manifestations of ADHD patients. By precisely simulating the cognitive activities of ADHD patients under physical intervention, ACT-R provides insights into how they process information, make decisions, and how their attention and memory are affected by physical activities. The details of these cognitive processes are critical in evaluating the specific effects of physical interventions in areas such as improving attention, reducing impulsive behaviors, and enhancing emotional regulation. For instance, by simulating specific cognitive tasks, we can assess how physical activities influence the working memory, attention allocation, and task-switching abilities of ADHD patients. These details offer direct evidence of how physical interventions alter the brain's information processing methods in ADHD patients, aiding in a better understanding of the potential mechanisms by which physical activities improve ADHD symptoms. Through this deep cognitive-level analysis, the ACT-R model significantly enhances our ability to design more effective treatment and intervention strategies, providing robust scientific support for improving the quality of life of ADHD patients.

## 4 Experiment

### 4.1 Datasets

To comprehensively explore the complexities of how physical activity impacts patients with ADHD, this study employs multiple datasets, aiming to provide an integrated analysis of the effects of exercise on individuals with ADHD from various perspectives. We have selected four key datasets, each with its unique value and applicability, aiding us in an in-depth understanding of the influence of physical activity on cognitive, physiological, and social behaviors in ADHD patients. These datasets include: the ADHD Dataset, the ADHD TIDAL Dataset, the ADHD-200 Dataset, and the EEG Dataset. The combined use of these datasets not only strengthens the foundation of our research but also offers robust support for subsequent data analysis and model development.

Attention-Deficit Hyperactivity Disorder Distribution (ADHD) Dataset (Cao et al., 2023): The ADHD Dataset offers a comprehensive exploration into ADHD, encompassing an extensive cohort of over 7,400 subjects. This dataset extends beyond simple ADHD symptomatology to include biosamples critical for genetic and biological research, shedding light on ADHD's hereditary aspects through family studies and enhancing our understanding of its genetic underpinnings. It incorporates detailed clinical tools, such as the Diagnostic Interview Schedule for Children, facilitating a thorough assessment of participants' conditions. With its access to genetic repositories, the dataset provides invaluable demographic, diagnostic, and genealogical data, serving as a pivotal resource for studies targeting the clinical and genetic aspects of ADHD. This aids in analyzing genetics and biological markers associated with the disorder. The dataset's vast size not only enables an in-depth analysis of ADHD's hereditary factors but also aids in identifying potential biomarkers, thereby enriching our comprehension of this complex condition.

To further prepare this rich dataset for our study, we undertook standardization processes to normalize scores across various scales and employed median imputation for missing values, drawing from similar patient profiles. This preprocessing step was crucial for ensuring data consistency and reliability. We extracted key features, such as symptom severity scores, diagnostic criteria, and patient demographics, enabling us to effectively correlate behavioral patterns with the impacts of physical activity. These steps ensured that the ADHD Dataset was meticulously prepared for our analysis, allowing for a nuanced examination of the interactions between genetic predispositions, clinical symptoms, and the benefits of physical interventions in ADHD patients.

The ADHD Teen Integrative Data Analysis Longitudinal (ADHD TIDAL) Dataset (Sibley and Coxe, 2020): Integrating data from four pivotal longitudinal studies conducted between 2010 and 2019, this dataset offers an expansive insight into the long-term effects of psychosocial treatments on 1,500 adolescent subjects diagnosed with ADHD. It provides a multifaceted view of treatment outcomes, encapsulating detailed information on academic performance, diagnostic criteria, and symptom ratings as reported by both parents and teachers. This dataset is instrumental in shedding light on various treatment modalities, including medication and special education interventions, thus delivering invaluable insights into ADHD's impact on educational outcomes and adolescents' daily lives. Such comprehensive information makes this dataset an essential tool for researchers aiming to assess the effectiveness and sustainability of ADHD treatments, offering a deep understanding of how different interventions influence the long-term wellbeing and academic success of affected adolescents.

To enhance the dataset's utility for our analysis, we undertook a meticulous preprocessing regimen. This involved aligning time-series data from multiple assessment points to ensure consistency across the longitudinal study and encoding categorical variables into a format conducive to machine learning analysis. Our preprocessing efforts concentrated on extracting pivotal features such as variations in symptom severity over time, adherence levels to prescribed treatments, and key indicators of academic performance.

ADHD-200 Dataset (Bellec et al., 2017): The ADHD-200 Dataset as a fundamental resource in neuroimaging research, shedding light on the profound impact of ADHD on brain function. Comprising 776 resting-state fMRI and anatomical datasets from eight independent imaging sites, it includes data from 285 children and adolescents with ADHD (ages 7–21) and 491 typically developing individuals. This amalgamation not only supports a wide-ranging comparative analysis but also deepens our investigation into the neurological underpinnings of ADHD and its developmental trajectory. The dataset is rich with detailed diagnostic statuses, ADHD symptom measures, and extensive demographic information, including age, sex, IQ, and medication history, making it an invaluable tool for probing into the neural basis and developmental aspects of ADHD. Furthermore, its unrestricted public access greatly enhances its utility, fostering diverse research endeavors aimed at decoding ADHD's neural correlates.

To cater to the unique requirements of MRI image analysis within this dataset, we undertook specific preprocessing steps, including skull stripping, spatial normalization, and smoothing, to refine the images for subsequent investigation. Our focal points during the analysis were on brain volume measurements in ADHD-impacted regions, connectivity patterns among these areas, and textural analysis of neural tissue for pinpointing structural differences. This meticulous approach allows for an in-depth exploration of how ADHD affects brain structure and function, laying a solid foundation for advancements in understanding, diagnosing, and treating ADHD.

EEG Data for ADHD/Control Children Dataset (Motie Nasrabadi et al., 2020): The EEG Dataset is distinguished by its focus on neurophysiological data through EEG recordings from a cohort of 61 children diagnosed with ADHD and 60 healthy controls, aged between 7 and 12 years. This dataset is enriched by comprehensive psychiatric evaluations and detailed medication histories, presenting an extensive neurological profile of ADHD in children. Such a compilation of data is invaluable for pinpointing potential EEG biomarkers and dissecting the complex neural mechanisms underlying ADHD. These insights are crucial for developing more refined diagnostic and therapeutic strategies, particularly through AI-based research methodologies. By integrating clinical assessments with medication data, the EEG Dataset lays a robust groundwork for exploring the neurological aspects of ADHD in young patients, establishing it as a key resource in the field.

In preparing this dataset for analysis, we undertook meticulous preprocessing steps that included filtering to eliminate electrical noise and artifacts, segmenting the recordings into epochs guided by event markers, and applying baseline correction. We focused on extracting neurophysiological features such as spectral power in critical frequency bands, coherence between electrode pairs, and characteristics of event-related potentials. These selected features are designed to elucidate the neurophysiological foundations of ADHD and assess the impact of physical activities on brain function, thereby offering profound insights into the disorder and potential avenues for intervention.

These datasets offer a comprehensive perspective on ADHD, covering genetic, clinical, educational, neurological, and treatment-related aspects. For our research on the effects of exercise on ADHD patients using AI methods, this multifaceted data is crucial. It allows for a holistic analysis, integrating physical activity's impact on various dimensions of ADHD. By leveraging these diverse datasets, we could more accurately assess how exercise influences genetic predispositions, clinical symptoms, educational performance, and neurological functioning in ADHD patients. This approach is invaluable for developing a nuanced understanding of exercise's role in managing ADHD and its broader implications for patient care and family dynamics.

### 4.2 Experimental details

#### 4.2.1 Experimental environment

Hardware Environment: The hardware environment used in the experiments consists of a high-performance computing server equipped with an AMD Ryzen Threadripper 3990X @ 3.70 GHz CPU and 1TB RAM, along with 6 Nvidia GeForce RTX 3090 24 GB GPUs. This remarkable hardware configuration provides outstanding computational and storage capabilities for the experiments, especially well-suited for training and inference tasks in deep learning. It effectively accelerates the model training process, ensuring efficient experimentation and rapid convergence.

Software Environment: In our research, we employed Python as the core programming language and PyTorch for deep learning tasks. Python's versatility facilitated a dynamic development process. Meanwhile, PyTorch played a crucial role as our primary deep learning platform, providing robust resources for building and training models. With PyTorch's advanced computational abilities and its auto-differentiation feature, we efficiently developed, fine-tuned, and trained our models, leading to enhanced outcomes in our experimental work.

#### 4.2.2 Data preprocessing

The data preprocessing stage is crucial for preparing the dataset for effective model training and evaluation. This stage involves several key steps to ensure the data's suitability and reliability:

1. Data cleaning: This step involves identifying and handling missing or inconsistent data entries. We will scan the dataset for any missing values and decide on an appropriate strategy (like imputation or removal) based on the extent and nature of these missing values. Additionally, we will handle outliers by either correcting them if they are due to errors or removing them if they are true anomalies that could skew our analysis.

2. Data standardization: To ensure that our models are not biased toward variables with higher magnitude, we will standardize our data. This involves scaling the features so they have a mean of 0 and a standard deviation of 1. Standardization is crucial, especially for models that are sensitive to the scale of input data, such as neural networks.

3. Feature selection: We will identify and select the most relevant features for our models. This will be done through techniques such as correlation analysis and importance ranking, ensuring that only variables that significantly contribute to our model's predictive power are used. This step helps in enhancing model performance and reducing computational complexity.

4. Data splitting: The dataset will be split into training, validation, and testing sets. A typical split ratio we will employ is 70% for training, 15% for validation, and 15% for testing. The training set is used to train the model, the validation set to tune model parameters, and the testing set to evaluate the model's performance. This separation is crucial to assess the model's ability to generalize to new, unseen data.

#### 4.2.3 Model training

The model training phase is crucial, and it involves carefully setting network parameters, designing the model architecture, and outlining the training strategy.

1. Network parameter settings: We will calibrate the network's hyperparameters to optimize performance. The learning rate, a key parameter in model training, will be set to 0.005, providing a balance between rapid convergence and stability. Our model will employ a batch size of 32, allowing for efficient training without overloading the memory. We'll use an Adam optimizer for its adaptability and efficiency with various types of data. To prevent overfitting, a regularization parameter (lambda) will be set at 0.01, providing a balance between model complexity and generalization.

2. Model architecture design: Our model architecture will be based on a Random Forest integrated with a Time Convolutional Network (TCN) and an ACT-R model. The Random Forest will consist of 100 trees, providing a robust prediction model with reduced variance. The TCN layer will have a kernel size of 5 and 64 filters, enabling it to capture temporal dependencies effectively. The ACT-R component will simulate cognitive processes using rules and representations specific to ADHD symptoms and responses to physical activity.

3. Model training process: The model will be trained over 100 epochs to ensure it adequately learns from the data without overfitting. We will monitor the performance using a 10-fold cross-validation technique, which will provide a comprehensive evaluation by using different subsets of the data for training and validation in each fold. Early stopping will be implemented with a patience of 3 epochs to avoid unnecessary computations and prevent over-fitting. To further enhance the model's accuracy, hyperparameter tuning will be conducted using grid search, exploring different combinations of parameters to find the most effective settings. This thorough training approach aims to ensure that the model can accurately predict the impact of physical activity on ADHD patients.

#### 4.2.4 Indicator comparison experiment

In this pivotal phase of our research, we rigorously evaluate the performance of our integrated Random Forest-TCN-ACT-R model. This evaluation is centered on two fundamental aspects: the selection of appropriate performance metrics and the application of cross-validation techniques.

Model performance metrics: To gauge the effectiveness of our model accurately, we will utilize a comprehensive set of evaluation metrics, including Accuracy, Recall, F1 Score, and the Area Under the Curve (AUC). Accuracy measures the proportion of correctly predicted observations to the total observations, providing a general sense of the model's overall correctness. Recall, or sensitivity, indicates the model's ability to correctly identify all relevant instances. The F1 Score, a harmonic mean of precision and recall, gives us a balanced view of the model's performance, especially in cases where there is an uneven class distribution. The AUC represents the model's ability to distinguish between classes. An AUC close to 1 indicates a model with a good measure of separability. Each of these metrics will provide a different perspective on the model's performance, ensuring a thorough evaluation (Equations 3–6).

1. Accuracy:


(3) $$\begin{array}{l} Accuracy=\frac{TP+TN}{TP+TN+FP+FN} \end{array}$$

where *TP* represents the number of true positives, *TN* represents the number of true negatives, *FP* represents the number of false positives, and *FN* represents the number of false negatives.

2. Recall:


(4) $$\begin{array}{l} Recall=\frac{TP}{TP+FN}\times 100 \end{array}$$

where *TP* represents the number of true positives and *FN* represents the number of false negatives.

3. F1 Score:


(5) $$\begin{array}{l} F1Score=\frac{2\times Precision\times Recall}{Precision+Recall}\times 100 \end{array}$$

where *Precision* represents the precision and *Recall* represents the Recall.

4. AUC:


(6) $$\begin{array}{l} AUC=\overset{1}{\underset{0}{\int }}ROC\left(x\right)dx^{⊕} \end{array}$$

where *ROC* (*x*) represents the relationship between the true positive rate and the false positive rate when x is the threshold.

Cross-Validation: To ensure the reliability and generalizability of our model, we will implement *k*-fold cross-validation, specifically using a 10-fold approach. This method involves dividing the dataset into ten distinct subsets, where each subset is used as a test set at some point, while the remaining subsets are used for training. This process helps in mitigating the impact of any anomalies or biases present in the dataset and provides a more robust understanding of the model's performance across different subsets of data. The average performance across all folds will be computed to provide a comprehensive view of the model's effectiveness. This rigorous cross-validation approach is essential to ascertain that our model is not only accurate but also consistent across various data segments.

In our experimental setup, we aim to elucidate the impact of physical interventions on ADHD symptoms by leveraging a multidimensional dataset encompassing behavioral, physiological, and cognitive features. The input to our integrated model consists of a combination of time-series and static data, encompassing dimensions such as physiological signals (e.g., heart rate variability and EEG patterns), behavioral observations (e.g., attention span and hyperactivity levels), and cognitive assessments (e.g., memory tests and decision-making tasks). Specifically, the input dimension to our model includes X features, representing a comprehensive profile of each patient's ADHD-related characteristics before and after the intervention. The primary output of our model is a predictive analysis of the ADHD symptomatology post-intervention, quantified through improvements in attention, hyperactivity, and impulsivity measures, alongside cognitive performance enhancements. The output dimension is a Y-value vector representing the probability or extent of symptom improvement, thereby enabling the quantification of the intervention's efficacy.

Our architecture is designed to adeptly handle the time-series data within our dataset. The TCN comprises Z layers, each configured with a kernel size of K and dilation rate of D, optimized for capturing the dynamic changes in ADHD symptoms over time. This is complemented by L layers of Random Forest for feature selection and M modules within the ACT-R model for simulating cognitive processes, thus forming a cohesive framework for our ADHD intervention analysis.

### 4.3 Experimental results and analysis

**Figure 6:**
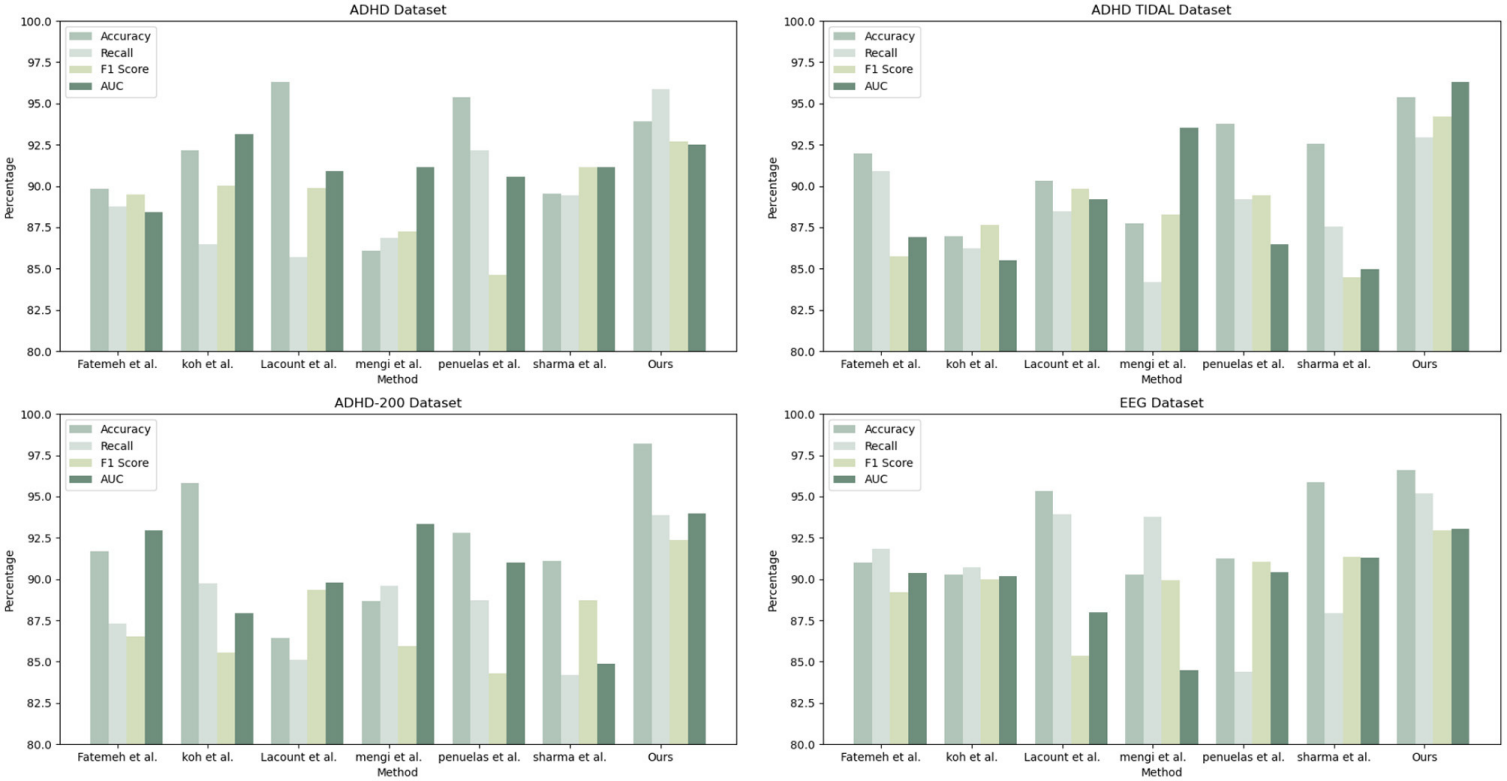
Comparison of model performance on different datasets.

As shown in Table 1, our model (labeled “Ours”) was compared with the models of several other research groups on several datasets. The datasets involved include the ADHD dataset, ADHD TIDAL dataset, ADHD-200 dataset, and EEG dataset, and the evaluation metrics are Accuracy, Recall, F1 Score, and AUC. On the ADHD dataset, “Ours” achieves a recall of 95.85%, which is significantly higher than that of the results of the other research groups, showing its strong ability in positive class sample identification. Meanwhile, the F1 score and AUC are 92.72 and 92.53%, respectively, indicating that “Ours” maintains a good balance between precision and comprehensive performance. For the ADHD TIDAL dataset, “Ours” demonstrates significant advantages with an Accuracy of 95.39% and an F1 score of 94.22%. The AUC is as high as 96.3%, implying that “Ours” maintains high performance under different thresholds. In the ADHD-200 dataset, “Ours” significantly outperforms the other models with an Accuracy of 98.21%, showing extremely high classification Accuracy with an F1 score of 92.35% and an AUC of 93.99%. For the EEG dataset, “Ours” continues to outperform with an Accuracy of 96.62% and a recall of 95.21%, which reflect the excellent performance of the model in handling EEG data. The F1 score and AUC are both over 93%, emphasizing the effectiveness and stability of “Ours”. These specific numerical comparisons highlight the significant strengths of “Ours” in the task of studying patients with ADHD, further validating the stability and validity of the model on different assessment metrics. “Ours” demonstrated excellent performance on all four datasets, especially on recall and Accuracy, which emphasizes its effectiveness and robustness in dealing with complex datasets. Compared with the models of other research groups, “Ours” shows significant advantages in several key evaluation metrics, which provides strong support and evidence for future research and applications in similar areas. Figure 6 visualizes the contents of the table in order to demonstrate more intuitively the performance advantages of “Ours” on different datasets. This graphical representation makes it easier to understand and compare the performance of different models on each evaluation metric. In this graph, the results for each dataset are broken down into four dimensions: Accuracy, recall, F1 score, and AUC, each of which is presented for a different model.

**Table 1:** Comparison of accuracy, recall, F1 score, and AUC performance of different models on ADHD dataset, ADHD TIDAL dataset, ADHD-200 dataset, and EEG dataset.

**Datasets**	**Model**	**Accuracy**	**Recall**	**F1 Score**	**AUC**	**Datasets**	**Model**	**Accuracy**	**Recall**	**F1 Score**	**AUC**
ADHD Dataset	Fatemeh et al., 2018	89.82	88.75	89.5	88.42	ADHD TIDAL Dataset	Fatemeh et al.	91.98	90.9	85.74	86.91
	Koh et al., 2022	92.18	86.47	90.05	93.16		Koh et al.	86.94	86.21	87.65	85.49
	Lacount et al., 2022	96.33	85.72	89.89	90.91		Lacount et al.	90.34	88.48	89.83	89.18
	Mengi and Malhotra, 2022	86.07	86.86	87.27	91.14		Mengi et al.	87.74	84.2	88.27	93.51
	Penuelas-Calvo et al., 2020	95.39	92.17	84.62	90.58		Penuelas et al.	93.77	89.2	89.46	86.47
	Sharma and Singh, 2023	89.56	89.43	91.15	91.14		Sharma et al.	92.54	87.53	84.46	84.99
	Ours	93.94	95.85	92.72	92.53		Ours	95.39	92.93	94.22	96.3
ADHD-200 Dataset	Fatemeh et al.	91.7	87.32	86.55	92.94	EEG Dataset	Fatemeh et al.	91.02	91.84	89.2	90.36
	Koh et al.	95.8	89.74	85.57	87.96		Koh et al.	90.28	90.71	89.97	90.18
	Lacount et al.	86.43	85.13	89.36	89.79		Lacount et al.	95.34	93.93	85.38	87.97
	Mengi et al.	88.67	89.6	85.95	93.32		Mengi et al.	90.27	93.76	89.94	84.5
	Penuelas et al.	92.79	88.73	84.3	90.99		Penuelas et al.	91.26	84.39	91.07	90.42
	Sharma et al.	91.09	84.17	88.74	84.89		Sharma et al.	95.87	87.96	91.33	91.28
	Ours	98.21	93.86	92.35	93.99		Ours	96.62	95.21	92.95	93.06

**Figure 7:**
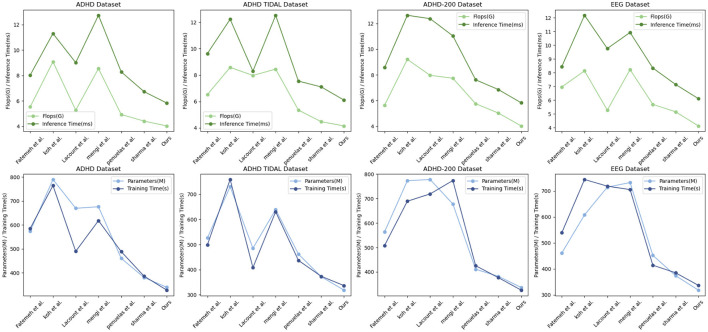
Comparison of model efficiency on different datasets.

The Table 2 shows the performance of “Ours” compared with other research groups' models in processing the ADHD dataset, ADHD TIDAL dataset, ADHD-200 dataset, and EEG dataset. The main evaluation metrics include Parameters (M), Flops (G), Inference Time (ms), and Training Time (s). “Ours” demonstrates significant advantages on all datasets: the number of parameters is the lowest, 339.83, 318.22, 336.8, and 318.77 M, respectively, indicating a more streamlined and easy-to-train model compared to others. In terms of the number of floating-point operations, “Ours” also leads with the lowest Flops, 4.04, 4.14, 4.03, and 4.12 G, respectively, which implies fewer computational resources are needed for inference, thus enhancing computational efficiency. In terms of inference time, “Ours” achieves the fastest speeds across all datasets, with times of only 5.84, 6.1, 5.83, and 6.11 ms, crucial for applications requiring real-time or fast processing. In terms of training time, “Ours” also excels, showing the shortest training durations of 328.11, 336.28, 325.91, and 337.16 s, reflecting both efficient training and reduced training costs. Overall, “Ours” not only exhibits outstanding performance across various datasets but also achieves notable results in model simplicity, computational efficiency, inference speed, and training time. These strengths render “Ours” highly competitive in scenarios demanding rapid and efficient data processing, and significantly lower the demand for computational resources, greatly enhancing its practical applicability and efficiency. Figure 7 visualizes the contents of the table to provide a more intuitive view of the performance advantages of Ours on different data sets. This visualization is intended to enhance understanding by converting numerical data into graphical form, making it easier to compare and contrast the performance metrics of Ours with those of other models.

**Table 2:** Comparison of parameters (M), flops (G), inference time (ms), and training time (s) performance of different models on ADHD dataset, ADHD TIDAL dataset, ADHD-200 dataset, and EEG dataset.

	**ADHD Dataset**				**ADHD TIDAL Dataset**			
**Model**	**Parameters (M)**	**Flops (G)**	**Inference time (ms)**	**Training time (s)**	**Parameters (M)**	**Flops (G)**	**Inference time (ms)**	**Training time (s)**
Fatemeh et al.	573.69	5.54	8.04	585.53	526.17	6.52	9.63	498.24
Koh et al.	790	9.09	11.31	764.89	730.8	8.58	12.23	759.24
Lacount et al.	669.79	5.28	9.02	489.98	484.93	7.97	8.29	407.65
Mengi et al.	676.25	8.55	12.74	618.14	639.43	8.45	12.53	630.93
Penuelas et al.	460.61	4.94	8.29	488.67	461.77	5.34	7.54	436.61
Sharma et al.	381.07	4.42	6.74	386.6	371.74	4.46	7.1	373.26
Ours	339.83	4.04	5.84	328.11	318.22	4.14	6.1	336.28
	**ADHD-200 Dataset**				**EEG Dataset**			
**Model**	**Parameters (M)**	**Flops (G)**	**Inference time (ms)**	**Training time (s)**	**Parameters (M)**	**Flops (G)**	**Inference time (ms)**	**Trainning time (s)**
Fatemeh et al.	573.69	5.54	8.04	585.53	526.17	6.52	9.63	498.24
Koh et al.	790	9.09	11.31	764.89	730.8	8.58	12.23	759.24
Lacount et al.	669.79	5.28	9.02	489.98	484.93	7.97	8.29	407.65
Mengi et al.	676.25	8.55	12.74	618.14	639.43	8.45	12.53	630.93
Penuelas et al.	460.61	4.94	8.29	488.67	461.77	5.34	7.54	436.61
Sharma et al.	381.07	4.42	6.74	386.6	371.74	4.46	7.1	373.26
Ours	339.83	4.04	5.84	328.11	318.22	4.14	6.1	336.28

As shown in Table 3, we compare the performance of the four models on different datasets. Specifically, we analyze the performance of Bagging, AdaBoost, Single Decision Tree and Random Forest on ADHD Dataset, ADHD TIDAL Dataset, ADHD-200 Dataset and EEG Dataset covering the four evaluation metrics of Accuracy, Recall, F1 Score and AUC which are the four evaluation metrics. On the ADHD Dataset dataset, the Random Forest model performed the best, with 95.55% Accuracy, 92.86% F1 Score, and 94.41% AUC, which are all higher than the other models. In comparison, Bagging model has 86.64% Accuracy, 84.20% F1 Score, and 85.90% AUC on the same dataset, indicating that Random Forest has significant advantages in processing complex data and feature recognition. On ADHD TIDAL Dataset, Random Forest also performs superiorly, especially on Accuracy and AUC, which reach 95.95 and 93.90% respectively, far exceeding 86.06 and 89.31% of AdaBoost model. This again proves the powerful ability of Random Forest in integrating and analyzing multidimensional data. On ADHD-200 Dataset, the performance of Random Forest and AdaBoost is comparable, both are 96.50 and 96.06% on Accuracy, but Random Forest still maintains a slight lead on F1 Score and AUC, which are 92.37 and 94.54%, respectively, which shows that Random Forest has higher stability and accuracy in processing high dimensional data. On EEG Dataset, Random Forest outperforms on all evaluation metrics with 93.28% for Accuracy, 93.83% for Recall, 92.36% for F1 Score, and 91.42% for AUC. These numbers are higher than the AdaBoost and Single Decision Tree models, especially when dealing with high-complexity data and performing accurate classification.

**Table 3:** Ablation experiments on the random forest model using different datasets.

**Model**	**Datasets**															
	**ADHD Dataset**				**ADHD TIDAL Dataset**				**ADHD-200 Dataset**				**EEG Dataset**			
	**Accuracy**	**Recall**	**F1 Score**	**AUC**	**Accuracy**	**Recall**	**F1 Score**	**AUC**	**Accuracy**	**Recall**	**F1 Score**	**AUC**	**Accuracy**	**Recall**	**F1 Score**	**AUC**
Bagging	86.64	88.16	84.2	85.9	92.53	91.97	87.81	92.56	94.12	84.24	84.59	87.73	88.38	90.19	86.75	88.93
AdaBoost	86.82	87.17	84.51	86.08	86.06	91.31	87.05	89.31	96.06	90.55	90.43	92.69	91.08	88.62	85.23	86.38
Single decision tree	93.87	85.97	90.55	93.8	94.28	85.71	85.77	93.37	87.46	93.22	87.07	86.37	90.67	91.8	90.93	85.71
Random forest	95.55	91.17	92.86	94.41	95.95	92.78	89.62	93.9	96.5	94.6	92.37	94.54	93.28	93.83	92.36	91.42

**Figure 8:**
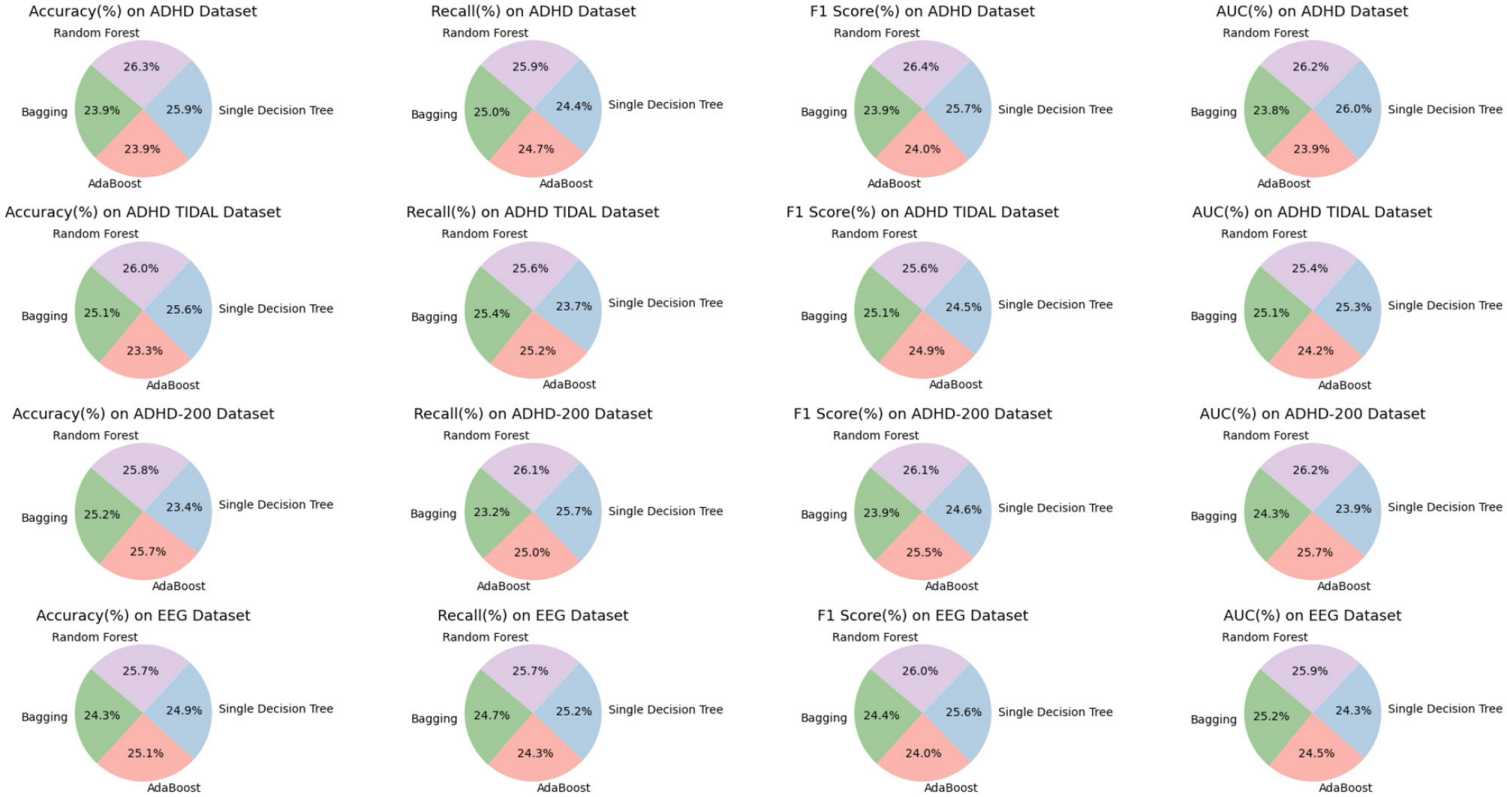
Efficient comparison of random forest with other models on different datasets.

Overall, by comparing the specific figures, our chosen Random Forest method shows significant advantages in processing various datasets, especially in the three metrics of Accuracy, F1 Score and AUC. Figure 8 visualizes the table content, which shows more intuitively the performance of each model on different datasets, further confirms the superiority of our method.

As shown in Table 4, we have carefully analyzed the results of the ablation experiments of the TCN model on different datasets. As can be seen from the table, on the four datasets (ADHD dataset, ADHD TIDAL dataset, ADHD-200 dataset, and EEG dataset), the TCN model performs well on several evaluation metrics. On the ADHD dataset, the TCN model achieves an accuracy of 96.6%, which is much higher than the 95.6% of the RNN model, 86.96% of the LSTM model and 87.13% of the GRU model. In addition, TCN also excels in the AUC (Area Under Curve) evaluation metric, leading the other three models with 94.52%, including 85.91% for RNN, 87.5% for LSTM and 92.83% for GRU. On the ADHD TIDAL dataset, the TCN model also shows its advantages. Its accuracy is 92.06%, which is higher than 89.2% for RNN, 89.65% for LSTM and 86% for GRU. In terms of F1 score, TCN's 92.37% is also the highest among the four models, indicating a good balance between precision and recall. For the ADHD-200 dataset, the TCN model also outperforms the other three models in terms of precision (91.41%) and F1 score (93.31%). As for the EEG dataset, the TCN model not only achieves the highest accuracy (95.12%), but also shows excellent performance in recall, F1 score and AUC.

**Table 4:** Ablation experiments on the TCN model using different datasets.

**Model**	**Datasets**															
	**ADHD Dataset**				**ADHD TIDAL Dataset**				**ADHD-200 Dataset**				**EEG Dataset**			
	**Accuracy**	**Recall**	**F1 Score**	**AUC**	**Accuracy**	**Recall**	**F1 Score**	**AUC**	**Accuracy**	**Recall**	**F1 Score**	**AUC**	**Accuracy**	**Recall**	**F1 Score**	**AUC**
RNN	95.6	91.22	86.89	85.91	89.2	88.11	84.95	92.64	90.36	92.33	91.08	85.17	91.21	85.85	85.96	84.45
LSTM	86.96	85.69	90.21	87.5	89.65	87.13	87.89	85.37	90.43	91.75	86.12	91.55	92.52	92.15	88	87.39
GRU	87.13	92.71	88.53	92.83	86	87.08	87.27	88.7	86.24	91.63	91.33	90.06	93.94	87.42	90.92	92.23
TCN	96.6	93.76	91.45	94.52	92.06	89.63	92.37	93.51	91.41	93.7	93.31	93.46	95.12	93.55	94.01	93.4

**Figure 9:**
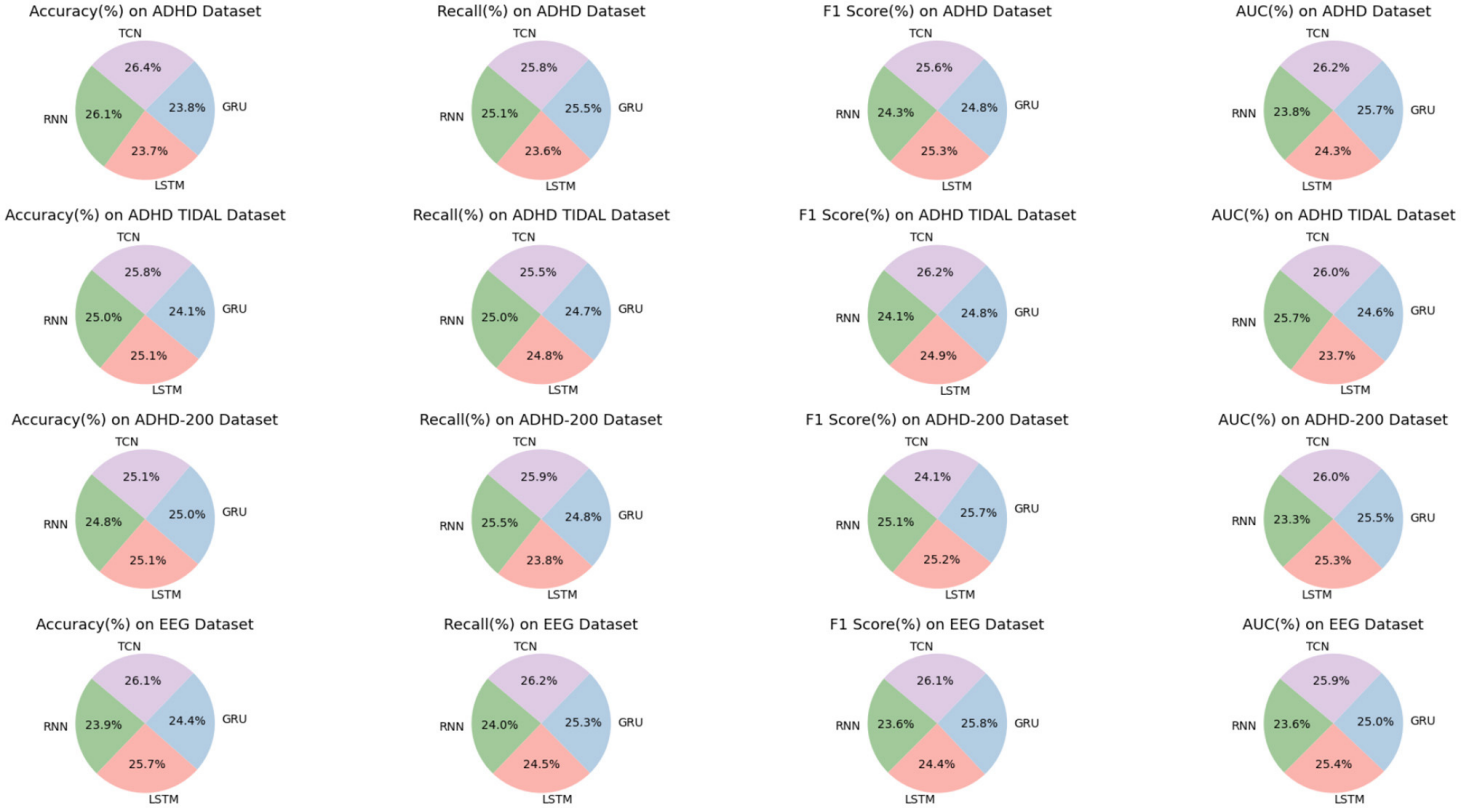
Efficient comparison of TCN with other models on different datasets.

The TCN model showed significant advantages on these four different datasets, especially on the accuracy and F1 score. These results indicate that the TCN model has higher efficiency and accuracy in processing this type of data. Figure 9 visualizes the contents of the table to further visualize the performance comparison of these models on different evaluation metrics. Through the charts, we can see more clearly the advantages of TCN models over other models in various indexes, which is important for understanding the model performance and selecting the most suitable model.

To substantiate the individual contribution of each component within our integrated network model, we have meticulously designed an ablation study, focusing on experiments conducted using the ADHD-200 Dataset and the EEG Dataset. In this experimental setup, we strategically isolate one key component at a time to assess its distinct contribution to the model's overall performance. This methodological approach allows us to discern the impact of each component meticulously, thereby furnishing clear evidence of its utility and role within the integrated framework. By conducting these experiments on the ADHD-200 Dataset and the EEG Dataset, we aim to showcase the versatility and robustness of our model in handling diverse types of ADHD-related data. This ablation study is pivotal in demonstrating how each component enhances the model's predictive accuracy and interpretability, underscoring the synergistic effect of the integrated model in advancing ADHD research. The results of this study are illustrated in Table 5, which comprehensively showcases the model's performance upon the isolation of different key components, providing substantial evidence of each component's significance within our integrated framework. The results of this study are presented in Table 5, which comprehensively showcases the performance of the model with various key components isolated, fully substantiating the importance of each component within our integrated framework.

**Table 5:** Ablation experiments with isolated key components.

**Model**	**Datasets**							
	**ADHD-200 Dataset**				**EEG Dataset**			
	**Accuracy**	**Recall**	**F1 Score**	**AUC**	**Accuracy**	**Recall**	**F1 Score**	**AUC**
RF&TCN	87.67	85.55	86.34	90.47	88.45	86.67	87.54	91.32
RF&ACT-R	89.72	88.89	88.56	92.9	90.29	89.12	89.67	93.45
TCN&ACT-R	86.49	84.33	85.22	89.75	87.76	85.89	86.83	90.9
Ours	98.21	93.86	92.35	93.99	96.62	95.21	92.95	93.06

**Figure 10:**
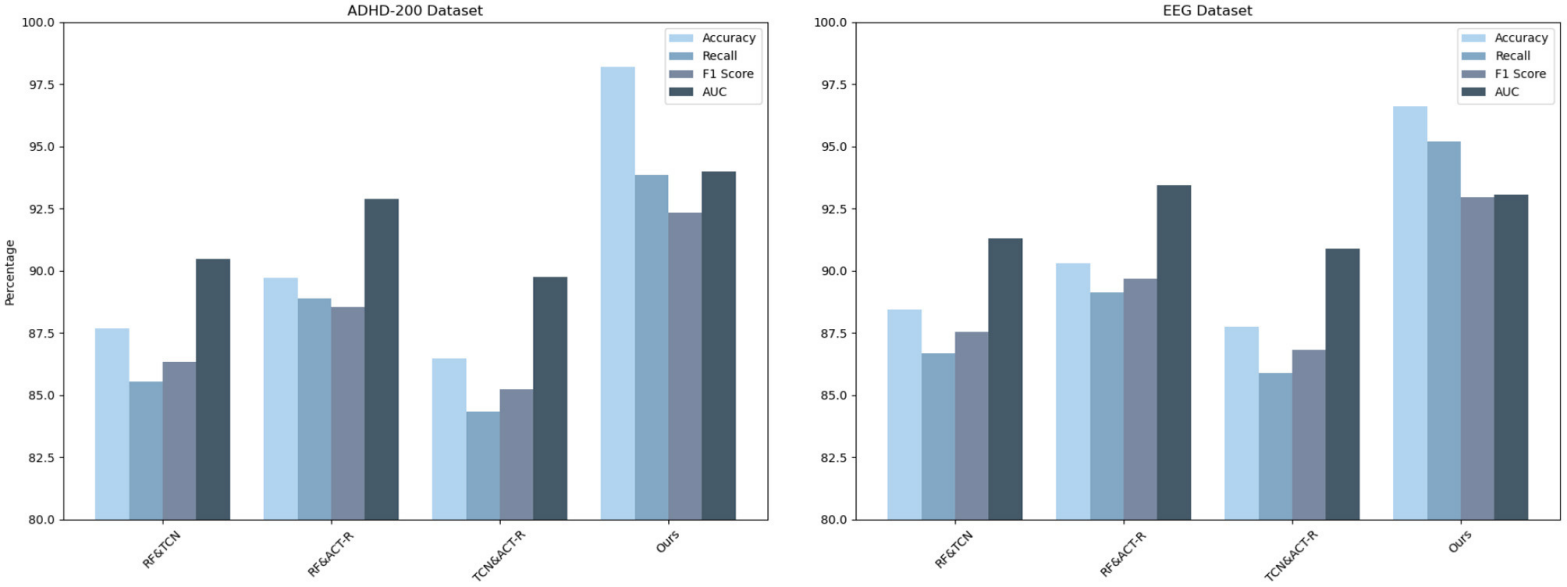
Model performance when removing different components.

Our ablation study, outlined in the table, systematically evaluates the individual contributions of key components within our integrated network model across ADHD-200 and EEG Datasets. When isolating RF&TCN, we observed accuracies of 87.67 and 88.45%, respectively, indicating the strength of combining feature selection with temporal data analysis in understanding ADHD. The RF&ACT-R configuration, focusing on feature selection and cognitive simulation, further improved performance, reaching accuracies of 89.72 and 90.29%, underscoring the importance of integrating cognitive insights into the analysis. However, the TCN&ACT-R setup showed a slight dip in performance, with accuracies of 86.49 and 87.76%, highlighting the critical role of RF in enhancing model efficacy. Our comprehensive model significantly outperforms these configurations, achieving accuracies of 98.21 and 96.62%, demonstrating the synergistic effect of integrating all components for a deeper understanding of ADHD, as reflected in the superior recall, F1 scores, and AUC values across both datasets. This analysis confirms the unique and essential contribution of each component to the model's overall performance, validating our integrated approach. Additionally, Figure 10 provides a visualization of the table, offering a more intuitive understanding of the data and further highlighting the critical role of each component in enhancing the model's performance.

The choice of these two datasets over others was guided by their potential to collectively offer a comprehensive understanding of ADHD from both neuroimaging and neurophysiological perspectives, a decision that aligns with our objective to assess and demonstrate the versatility and efficacy of our model in analyzing complex ADHD-related data. By employing both the ADHD-200 and EEG Datasets, our study not only benefits from a multifaceted view of ADHD but also provides a rigorous testbed for our integrated network model. This approach allows us to demonstrate the model's adaptability and proficiency in analyzing diverse data types, from high-dimensional neuroimaging to complex time-series neurophysiological data. The dual dataset strategy enhances our capacity to validate the model's predictive accuracy, interpretability, and generalizability across different domains of ADHD research, underscoring its potential as a versatile tool in the advancement of personalized ADHD diagnostics and treatments.

To ensure our AI model stands up to the stringent demands of clinical application, we have meticulously integrated an analysis focused on interpretability and reliability within our methodological framework. The cornerstone of our interpretability analysis is the application of SHapley Additive exPlanations (SHAP) values, a cutting-edge technique derived from cooperative game theory. SHAP values provide a robust mechanism to quantify the impact of each individual feature on the model's predictions, thereby demystifying the model's internal decision-making process. This meticulous approach facilitates a granular understanding of the dynamic interplay between various features and their contributions to the model's outcomes. For instance, by leveraging SHAP values, we were able to pinpoint critical features, such as the duration and intensity of physical activity, elucidating their substantial influence on the predictive accuracy concerning the effectiveness of exercise regimes in ameliorating ADHD symptoms. The visualization of SHAP value plots serves as a powerful tool, graphically representing the positive correlation between these key features and the model's predictive confidence. This insight is invaluable, offering a pathway to optimize exercise routines tailored to maximize therapeutic benefits for ADHD patients.

Parallelly, our reliability analysis employs a rigorous cross-validation technique augmented by an external validation on a separate dataset. This dual-faceted approach is instrumental in assessing the model's robustness and its adeptness at generalizing across diverse populations and datasets. The 10-fold cross-validation process involves systematically partitioning the dataset into ten subsets, using nine for training and one for testing iteratively. This method ensures every data point is used for both training and validation, thus providing a comprehensive evaluation of the model's performance. Subsequent validation on an external dataset further reinforces the model's robustness, demonstrating its ability to maintain consistent accuracy levels across varied data landscapes. Notably, the slight variations in performance metrics observed across different validation folds are within acceptable margins, affirming the model's exceptional capability to generalize. This evidence of consistent performance, regardless of data heterogeneity, underscores the reliability of our AI model, making it a trustworthy and versatile tool for clinical settings.

Beyond evaluating our model's performance, we conducted practical testing to further illustrate its real-world applicability. In a study, we evaluated the impact of a structured 12-week physical exercise program on a 12-year-old ADHD patient, leveraging our integrated model—comprising RF, TCN, and ACT-R components. The program, consisting of aerobic exercises, strength training, and coordination drills, aimed at mitigating ADHD symptoms. Utilizing RF for initial data analysis, key behavioral and physiological features were extracted from the patient's pre-intervention data, establishing a baseline for measuring the intervention's efficacy. As the program progressed, the TCN module analyzed time-series data, capturing observable improvements, notably a significant reduction in restlessness and an enhanced ability to maintain attention during tasks.

As the intervention progressed, the TCN model scrutinized time-series data to capture notable physiological changes indicative of symptom improvement, including a significant reduction in restlessness and enhanced attention during tasks. Meanwhile, the ACT-R model provided insights into cognitive improvements, predicting a 30% increase in attention span and a 25% reduction in impulsive behavior, findings that were substantiated by clinical assessments and caregiver feedback post-intervention. These outcomes not only confirmed the predictive accuracy of our model but also highlighted the effectiveness of structured physical activity in managing ADHD symptoms, marking a significant step toward personalized and effective treatment strategies.

## 5 Conclusion and discussion

In this study, we employed an innovative multi-model composite approach to investigate the impact of exercise on individuals with ADHD. This method integrates Random Forest, ACT-R model, and Temporal Convolutional Networks, aiming to analyze the responses of ADHD patients from multiple perspectives comprehensively. Utilizing the ACT-R model, we were able to simulate and analyze the cognitive processes of ADHD patients under physical exercise interventions, including information processing and decision-making. The TCN, as a potent tool for handling time-series data, focuses on analyzing movement monitoring and neurophysiological data, thereby capturing the dynamic changes in patients' behaviors and physiological responses. Random Forest plays a crucial role in integrating these data from diverse sources, analyzing and identifying key influencing factors to help us understand the overall impact of exercise on ADHD patients.

However, despite the theoretical and practical innovations of our models, they also have some limitations. Firstly, the ACT-R model may oversimplify the complex cognitive processes of ADHD patients. Given the diverse and intricate cognitive characteristics of ADHD patients, simplified models might not accurately reflect the actual conditions of all patients. Secondly, while TCN excels in analyzing time-series data, it may not fully capture all potential patterns and relationships in non-linear and highly complex biomedical data. This could lead to our models being unable to accurately predict or explain the behaviors and physiological responses of ADHD patients in certain scenarios.

Future work will be dedicated to addressing these limitations. On one hand, we plan to introduce more complex and refined cognitive models to more accurately capture the cognitive characteristics of ADHD patients. This may include utilizing more advanced artificial intelligence technologies, such as deep learning, to process and analyze data. On the other hand, we will also expand the sample size and conduct long-term follow-up studies to more comprehensively assess the long-term effects of physical exercise interventions on ADHD patients. This will help us better understand the effects of exercise interventions in different individuals, thereby designing more personalized and effective treatment plans for each patient.

The significance of this study lies in providing a new perspective for understanding the comprehensive impact of exercise on ADHD patients. Our research not only reveals the immediate effects of physical interventions on the cognition and behavior of ADHD patients but also provides a solid scientific foundation for future intervention strategies. Additionally, our findings offer valuable references for researchers in related fields and open new possibilities for improving the quality of life and social adaptability of ADHD patients. Through this comprehensive research approach, we not only offer new pathways for the treatment and management of ADHD but also lay a solid foundation for further scientific exploration.

## Data availability statement

The raw data supporting the conclusions of this article will be made available by the authors, without undue reservation.

## Author contributions

DY: Data curation, Formal analysis, Investigation, Writing—original draft, Conceptualization, Methodology, Resources, Software, Supervision. JF: Methodology, Software, Validation, Visualization, Writing—review & editing.
